# Using best-worst scaling choice experiments to elicit the most important domains of health for health-related quality of life in Singapore

**DOI:** 10.1371/journal.pone.0189687

**Published:** 2018-02-08

**Authors:** Elenore Judy B. Uy, Dianne Carrol Bautista, Xiaohui Xin, Yin Bun Cheung, Szu-Tien Thio, Julian Thumboo

**Affiliations:** 1 Department of Rheumatology & Immunology, Singapore General Hospital, Singapore, Singapore; 2 Singapore Clinical Research Institute, Singapore, Singapore; 3 Centre for Quantitative Medicine, Duke-NUS Medical School, Singapore, Singapore; 4 Academic Clinical Programme for Medicine, Singapore General Hospital, Singapore, Singapore; 5 Tampere Center for Child Health Research, University of Tampere and Tampere University Hospital, Tampere, Finland; 6 Office of Clinical, Academic & Faculty Affairs, Duke-NUS Medical School, Singapore, Singapore; 7 Yong Loo Lin School of Medicine, National University of Singapore, Singapore, Singapore; National University of Singapore, SINGAPORE

## Abstract

Health-related quality of life (HRQOL) instruments are sometimes used without explicit understanding of which HRQOL domains are important to a given population. In this study, we sought to elicit an importance hierarchy among 27 HRQOL domains (derived from the general population) via a best-worst scaling survey of the population in Singapore, and to determine whether these domains were consistently valued across gender, age, ethnicity, and presence of chronic illnesses. We conducted a community-based study that sampled participants with quotas for gender, ethnicity, age, presence of chronic illness, and interview language. For the best-worst scaling exercise, we constructed comparison sets according to a balanced incomplete block design resulting in 13 sets of questions, each with nine choice tasks. Each task involved three HRQOL domains from which participants identified the most and least important domain. We performed a standard analysis of best-worst object scaling design (Case 1) using simple summary statistics; 603 residents participated in the survey. The three most important domains of health were: “the ability to take care of self without help from others” (best-worst score (BWS): 636), “healing and resistance to illness” (BWS: 461), and “having good relationships with family, friends, and others” (BWS: 373). The 10 top-ranked domains included physical, mental, and social health. The three least important domains were: “having a satisfying sex life” (BWS: -803), “having normal physical appearance” (BWS: -461), and “interacting with others (talking, shared activities, etc.)” (BWS: -444). Generally, top-ranked domains were consistently valued across gender, age, ethnicity, and presence of chronic illness. We conclude that the 10 top-ranked domains reflect physical, mental, and social dimensions of well-being suggesting that the sampled population’s views on health are consistent with the World Health Organization’s definition of health, “a state of complete physical, mental and social well-being and not merely the absence of disease or infirmity”.

## Introduction

Many instruments have been developed, validated, and used to measure health-related quality of life (HRQOL). Generic health status instruments that can be used among people without illness and among different patient groups are widely used; such instruments allow health state comparisons and valuations to guide healthcare decisions at the individual and population level. These include the instruments from the Patient-Reported Outcomes Measurement Information System (PROMIS), the World Health Organization Quality of Life (WHOQOL) group, Short Form 36 (SF-36) of the Medical Outcomes Study, and the EuroQOL five-dimension questionnaire(EQ-5D), to name a few. Despite the popularity of these instruments, there are notable limitations: First, most of these instruments have been developed and used in Western populations and, only later, adapted for use in other populations. The WHOQOL, having been developed in multiple countries worldwide from its inception, is a notable exception [1–4]. Second, despite painstaking efforts to cross-culturally adapt these instruments prior to their use in other countries, there is mounting evidence that these HRQOL instruments do not adequately account for the differences in culture, health experiences, and priorities in other populations, most especially in Asian countries [5–12]. In addition, these instruments were often developed using a “top-down” approach, with the published literature and the experts in the field guiding how HRQOL is conceptualized and which domains are developed into instruments. This is also evident among HRQOL instruments developed in Asia [13–15].The process of developing HRQOL instruments is at times carried out without formal inquiry into which domains are of importance to the patients and the members of a given population, the “end-users” who are envisioned to use the developed HRQOL instruments. Patient input is sometimes lacking at the point of framework development and identification of domains to be developed into an instrument, and is often limited to reviewing adequacy of domain coverage and the wording of items [1–4].

To address these gaps, we sought to determine the domains of health that are important to Singaporeans by using a “ground-up” approach. With a population of 3.87 million citizens and permanent residents, Singapore is a microcosm of Asia—a multi-cultural, multi-ethnic country with individuals of Chinese, Malay, and Indian ethnicities communing with each other and using English as the lingua franca [16]. Developing an HRQOL instrument in this setting provides a unique opportunity to explore cultural variation in conceptualizations of health and develop a unified instrument to measure HRQOL in this population.

In a previous qualitative study based on focus group discussions and in-depth interviews, we developed a framework to capture the domains of health which are important to the multi-ethnic population in Singapore [17]. The framework identified 27 HRQOL domains. In the current study we sought to determine which of these 27 health domains are perceived to be the most important to subjective wellbeing, from the point of view of the population in Singapore, and to determine whether these domains are consistently valued across gender, age, ethnicity, and presence of chronic illnesses.

## Methods

This study underwent ethical review and was approved by the Singhealth Centralised Institutional Review Board.

### Study design

The best-worst scaling survey was conducted using a cross-sectional study design. To elicit the “importance ranks” among 27 HRQOL domains for the Singapore population, we administered a best-worst scaling (BWS) survey to sampled, community-dwelling participants. Singapore citizens or permanent residents, 21 years or older, of Chinese, Malay or Indian ethnicity, who spoke either English or Chinese (Mandarin) were eligible.

### Creating linguistically-simple domain definitions for ranking

We previously identified 27 health domains that spanned physical, mental, and social health using focus groups and in-depth interviews with Singapore citizens and permanent residents [17]. To standardize interpretation and facilitate comprehension of the health domains during ranking, we used the transcripts from the previous study to develop linguistically-simple English definitions for the 27 domains. To test the comprehensibility of the English domain definitions, we conducted cognitive interviews with convenience-sampled participants of varying age, level of education, gender, and ethnicity from the specialist outpatient clinics of the Singapore General Hospital. The domain definitions were revised iteratively based on inputs from the cognitive interviews. Each iteration was based on the input from three to four cognitive interview participants. Thereafter, we developed a Chinese version of the domain definitions through a forward and back translation process [18]. As in the English version, we performed cognitive interviews with native Chinese speakers and revised the wording of the definitions accordingly. Participants ranked the domains based solely on these definitions; no domain labels or other concept clarifications were provided either verbally or in writing.

### Best-worst scaling (BWS)

We employed “object case” or “Case 1” BWS to determine the importance hierarchy of the 27 health domains. In BWS, the best and worst choices represent extremes of an underlying latent or subjective dimension of interest. In a Case 1 BWS task, participants are asked to identify which of a fixed set of “objects” they find the best (“most attractive”, “most useful”, or “most important”) and the worst (“least attractive”, “least useful”, or “least important”) [19, 20]. The mathematical models and proofs for Case 1 BWS are provided in Marley and Louviere [21].

In this study, the BWS exercise had 27 “objects”. Each health domain, represented by a linguistically-simple domain definition, was one “object”.

#### Construction of comparison sets

Our goal was to elicit the three highest valued domains among 27 HRQOL domains for the Singapore population as a whole, and within subgroups of gender, age, ethnicity, and presence of chronic illness. We used the algorithm of Khare and Federer [22] to construct comparison sets based on a balanced incomplete block design (BIBD) for 27 objects generating 13 versions (replicates), each containing nine choice tasks (blocks) of size three (i.e., three health domains per choice task) (S1 Table). In this particular BIBD, each health domain appears 13 times and every pair of health domains appears once (concurrence of one). The position of the item in a choice set (i.e., 1^st^, 2^nd^, or 3^rd^ from the top of the page) is rotated across the 13 versions with each item appearing 4 to 5 times in each position. Each participant completed two versions, randomly selected, without replacement from the universe of 13 versions. Hence, each participant completed 18 choice tasks in total. In each choice task, participants were asked to identify the most important and the least important health domain. The most important health domain was coded as “1” and the least important as “-1”. We piloted and refined the BWS survey instrument with a convenience sample of participants from the community (n = 20) and specialist outpatient clinics (n = 45).

There are various challenges to obtaining rankings and ratings for a large set of conceptually complex items. In this situation, BWS has various advantages over standard methods of ranking or rating a full set of items. Considerable cognitive burden is involved in ranking a large set of concepts. This makes simply ranking a full list of items unlikely to yield useful information. By presenting only a manageable number of items at any one time (at most 4 or 5 items), BWS provides item ranks across a full set of items. By rating each item, BWS scaling provides information on the magnitude of the importance of each of the items, but also provides information on the importance of the items relative to each other (i.e., it minimizes situations where all items receive equal ratings suggesting equal magnitude of importance even when this is not truly the case) [23,24]. The BWS approach also minimizes limitations associated with numeric rating scales such as the influence of culturally-dependent differences in the interpretation of rating scales and/or personal response styles [18–20], or the risk of acquiescent response bias, which is the tendency to agree rather than disagree regardless of item content. Across a sampled population, the design of choice sets also sets the number of times that each item appears alongside each of the other items and rotates the position of an item so that it appears a set number of times in each position. These limit the influence of the way in which items are presented (i.e., groupings in the set and item position) on the resulting item rank. Despite these advantages, BWS also has limitations: 1) although the rotation of items and item positions minimizes the influence of item presentation on the resulting item ranks, ranking a large set of items using an exhaustive BWS design could mean presenting an unfeasibly large number of choice sets to respondents, 2) respondents are only able to rank subsets of items at any one time without considering the entire set of items to be ranked–whether or not respondents would interpret and rank full sets of items similarly is not clear.

#### Sampling of participants for community-based survey

We commissioned a survey company, Nielsen, to recruit participants, administer the survey, and collect survey data.

Participant selection was made according to a multi-stage, sampling plan. The survey company used a proprietary sampling frame that grouped households into geographical clusters, which served as primary sampling units. The sampling frame, comprised of 825,000 households, represented 80% of the non-condominium/non-apartment residential households in Singapore (88% of Singapore residential households are non-condominium/non-apartment dwelling types). Due to access difficulties into condominiums and apartments (12% of Singapore residential households), these gated communities were excluded from the survey. The profiles of the individuals residing in the excluded dwelling types were similar to those who were residing in landed properties which were included in the survey sampling frame [17].

The first stage of sampling involved a simple random sample to select 39 primary sampling units. The second stage of sampling involved a systematic sample to select households; interviewers went door-to-door using fixed route rules and skip patterns to identify candidate households for inclusion. Within each identified household, a single, eligible participant was selected for interview (third stage sampling).

To ensure representation of the demographic segments of interest, we implemented quotas based on gender, age, ethnicity, presence of chronic conditions, and language of interview. The list of chronic conditions used for specifying quotas was based on the Singapore Burden of Disease Study (2010) [25].

#### Data collection via community-based survey

Interviewers underwent a preparatory briefing to harmonize the understanding of concepts (e.g. study objectives and definition of terms in the survey instrument) and standardized criteria and/or methods pertinent to participant selection, administration of the best-worst scaling exercise using standardized show cards, and entry of data into a handheld data capture system. Interviewers went door-to-door and collected all data directly from the survey participant. Interviews were conducted from March 21 to May 11, 2015, 10:00 AM to 10:00 PM, daily; with up to three visit attempts (first attempt with two follow-up visits, at least one follow-up visit conducted either in the evening or the weekend) for each identified household.

In the BWS exercise, prior to being shown each choice set, participants were asked the question “Which ONE is the most important for you to be happy and satisfied with life?”. After indicating a choice, participants were then asked “Which ONE is the least important?”. To ensure that participants understood the mechanics of the ranking exercise, the interviewers administered sample choice sets before proceeding to the actual BWS survey instrument. The instructions for the BWS exercise are simple and remain the same throughout the 18 choice sets. As such, the survey did not require an interview guide. However, interviewers were equipped with standardized show cards which showed the two stem questions and one choice set (containing 3 choices) printed in large font, on A4-sized paper (S1 Fig). Interviewers were instructed to read the questions and choices exactly as shown in the show card and were not allowed to reword, or expound on the stem questions and the items in the choice sets.

Fifteen interviewers carried out the original survey. With the exception of one interviewer who only recruited one participant, each interviewer recruited 45 to 48 participants. We tested for interviewer effects by comparing the proportion of participants who were unable to rank (UTR) a number of choice sets: 0, 1, 2, …, up to 18 (the total number of choice sets). We called this the interviewer-specific UTR distribution. We formally tested (using the Kruskal-Wallis non-parametric test) for comparability of the UTR distributions across 14 interviewers and identified one interviewer with an extreme distribution, for whom 100% of participants were unable to rate all 18 choice sets. After identifying a breach in the interview protocol, we deemed the data to be invalid and asked for a full replacement sample. The recruitment of replacement participants was approved by the institutional review board and was carried out from May 9 to 19, 2016 by two interviewers who were involved in the original survey. Each replacement respondent was matched, based on age, gender, and ethnicity, to an original participant.

#### Statistical analysis

We followed the standard analysis of best-worst object scaling designs (Case 1) which is based on the best-worst score (B-W) score, as presented by Louviere and colleagues [26]. The B-W score of a health domain is obtained by subtracting the number of times the domain was chosen as least important from the number of times it was chosen as most important across participants. Positive values of the B-W score indicate that a health domain was chosen more frequently as most important, and negative values indicate that a health domain was chosen more frequently as least important. We calculated the average B-W score by dividing the B-W score by the number of participants as all 27 health domains appear with a frequency of two in each participant interview.

Another descriptive measure of importance is the ratio score derived by taking the square root of the total best score divided by the total worst score, i.e., $\sqrt{B/W}$. The resulting coefficient indicates the choice probability relative to the most important domain [21, 27–30]. To facilitate interpretation and comparison across health domains, the square root of (B/W) for all health domains was then scaled by a factor equal to the maximum square root of (B/W) so that the most important health domain was indexed at 100. For simplicity, these scaled quantities are referred here onwards as “index values”. Higher index values represent greater importance ranks.

To further ascertain if choices were consistent across participants, we calculated the standard deviation (SD) of B-W scores and the coefficient of variation (CV) (ratio of the SD to the average B-W score). Higher absolute values for the coefficient of variation represent greater choice heterogeneity.

To compare the top-ranked health domains across subgroups of gender, age, ethnicity, and presence of chronic illnesses, we used a combination of the index value and the CV. Higher index values combined with smaller CV values imply that there is a high concurrence within a group in the choice of a particular health domain as the most important; smaller index values combined with smaller CV values imply a high concurrence within the group in the choice of a particular domain as the least important.

As a sensitivity analysis, we estimated an exploded logit model [31] to compare the importance ranks using this method with those obtained using the standard analysis. We utilized the B-W score of the health domain (at the participant level) as the dependent variable and a set of 26 dummy variables as independent variables, one for each health domain with the domain “self-care” (#14) as reference class. We used the SAS PHREG procedure to output the regression coefficients (betas) and odds ratio. The beta estimate and odds ratio associated with “self-care” were 0.0 and 1.0 respectively. Health domains with smaller betas or smaller odds ratio are associated with a higher standing in the importance hierarchy. To verify agreement (i.e., consistency) between the two ranking approaches, we computed Pearson’s correlation coefficient between the domains’ total B-W score and the beta estimates. A correlation coefficient with absolute value equal to or greater than 0.90 was deemed acceptable to confirm agreement.

## Results

### Creating linguistically-simple definitions for the domain-ranking exercise

The demographic characteristics of the English and Chinese language cognitive interview participants are summarized in Table 1. To test comprehensibility of the English definitions, we conducted cognitive interviews with convenience-sampled English-speaking participants (n = 29) from the specialist outpatient clinics of the Singapore General Hospital. This resulted in revisions to the wording of the English definitions, which were finalized after five iterations. Thereafter, we developed a Chinese version of the domain definitions through a forward and back translation process [18]. As in the English version, we performed cognitive interviews with native Chinese speakers (n = 12) and revised the wording of the definitions accordingly. We finalized the wording of the Chinese domain definitions after four iterations. The set of 27 health domains and corresponding definitions, as presented to participants in the English-language survey, is given in Table 2.

**Table 1:** Socio-demographic characteristics of English and Chinese cognitive-interview participants.

	English-language cognitive interview (n = 29)		Chinese-language cognitive interviews (n = 12)	
Age (mean, range)	47.00 (SD:12.18, Range: 30 to 77)		48.33 (SD: 19.61, Range: 22 to 75)	
	Frequency	Percentage (%)	Frequency	Percentage (%)
Age group*				
21 to 34 years	6	20.7	4	33.3
35 to 49 years	8	27.6	1	8.3
50 years and older	15	51.7	7	58.3
**Gender**				
Female	16	55.2	9	75.0
**Ethnicity**				
Chinese	15	51.7	12	100.0
Malay	7	24.1	-	-
Indian	7	24.1	-	-
**Marital Status**				
Single	5	17.2	6	50.0
Married (or living as married)	18	62.1	5	41.7
Separated or Divorced	4	13.8	1	8.3
Widowed	2	6.9	-	-
**Years of Education**				
0 to 6 years	1	3.5	3	25.0
7 to 12 years	14	48.3	6	50.0
≥13 years	14	48.3	3	25.0

**Table 2:** Definition of the domains ranked in the community-based survey.

Domain name		Domain definitions*
**Physical Health**		
**1**	Physical appearance	Having normal physical appearance (acceptable weight, looking healthy).
**2**	Vitality	Having energy to do things.
**3**	Physical fitness and mobility	Being able to carry out physical activities and move around without difficulty.
**4**	Healing and resistance to illness	Not falling sick easily and getting well quickly when you are sick.
**5**	Breathing	Being able to breathe well (e.g. no nose blockage, no asthma attacks).
**6**	Eating and digestion	Being able to eat and digest food well.
**7**	Bowel movement	Being able to pass motion regularly (not having constipation).
**8**	Bladder control	Being able to control your urine.
**9**	Sex	Having a satisfying sex life.
**10**	Sleep	Being able to sleep well.
**11**	Eyesight	Having good eyesight.
**12**	Hearing	Being able to hear well.
**13**	Speech	Being able to speak clearly so that others can understand (e.g. no slurring of speech).
**14**	Self-care	Being able to take care of self-care needs without help from others (i.e. eating, bathing, getting dressed).
**15**	Discomfort and pain	No aches or pains in the body.
**Mental Health**		
**16**	Emotions	Being happy. Not sad, angry, or worried.
**17**	Self-esteem	Having confidence in yourself.
**18**	Personal freedom	Being independent and in control of your life.
**19**	Sense of growth	Being able to improve in knowledge, skills, and emotional maturity.
**20**	Engagement with life	Being able to find satisfaction and meaning in the things that you do.
**21**	Resilience	Being able to overcome difficulties in life.
**22**	Mindset	Thinking positively in life.
**23**	Active mind	Being mentally active (e.g. reading, learning new things, taking up a new hobby).
**24**	Memory	Having a good memory (able to remember objects, thoughts, and events)
**Social Health**		
**25**	Social contact	Interacting with others (e.g. talking, shared activities, etc.).
**26**	Social relationships	Having good relationships with family, friends and others.
**27**	Social role	Having a role in the lives of others and being able to fulfil that role (e.g. being a good employee, a good wife, a good son).

*Only the domain definitions were shown to the respondents of the domain-ranking survey

### Participant eligibility and response rate

We pre-selected and approached 7591 households; the residents of 4013 (53%) households did not answer the door despite repeated callbacks; 2896 (38%) households had residents who fit the survey inclusion criteria. Of these 2896 households, 92% had residents who were willing to participate in the survey. However, only 603 (21%) households were actually able to participate in the survey due to full quotas or other logistic challenges (e.g. resident was unavailable during the interview appointment). Interviews lasted a median of 29 minutes and ranged from 8 to 174 minutes.

At first glance, the wide range in the duration of interviews appears alarming. However, data from the cognitive interviews and pilots conducted at the start of this study showed that highly literate participants could complete the repetitive BWS exercise within a short period of time. Also, interviews were carried out in the respondents’ homes. As such, many respondents may have had interruptions in the course of the interview (e.g. children requiring attention). However, the electronic data capture system used by the survey company does not allow the timer to be paused during such interruptions, which could have led to artificially prolonged interview duration. In addition, the survey recruited participants of all ages; elderly patients (in both English and Chinese surveys) tended to have longer interview durations.

#### Socio-demographic characteristics of sampled population

Six hundred and three (n = 603) residents participated in the BWS survey. Table 3 summarizes the socio-demographic attributes of the survey participants included in the analysis (555 participants from the original sample, 48 participants recruited as replacements). The mean age of the respondents was 45.4 years (SD: 16.3, range: 21 to 88). Many participants reported having “personally had any significant experience with poor health or illness” (17.1%), having a “close friend or family member who had poor health or illness” (39.1%), or having “provided care to a close friend or family who had a chronic illness” (17.7%). Of those who reported having provided care to a close friend or family member (n = 107), 38.3% reported that they were the primary caregiver.

**Table 3:** Socio-demographic characteristics of BWS survey participants (n = 603).

	Sampled Population		
	Frequency	Percentage (%)	Singapore Population 2014 (%) [32]
Age group*			
**21 to 34 years**	198	32.8	21.2
**35 to 49 years**	176	29.2	23.9
**50 years and older**	229	38.0	32.7
Gender*			
**Female**	307	50.9	50.8
Ethnicity*			
**Chinese**	301	49.9	74.3
**Malay**	150	24.9	13.3
**Indian**	152	25.2	9.1
Language of interview*			
**Chinese**	150	24.9	n.a.
**English**	453	75.1	n.a.
Chronic illness*			
Present	301	49.9	n.a.
**Chronic disease condition (self-reported)**			
**Anxiety disorder**	3	0.5	n.a.
**Asthma**	41	6.8	n.a.
**Cancer**	0	0.0	n.a.
**Chronic Obstructive Pulmonary Disease (COPD)**	2	0.3	n.a.
**Depression**	10	1.7	n.a.
**Diabetes**	70	11.6	n.a.
**Adult-onset Hearing loss**	3	0.5	n.a.
**Heart disease**	1	0.2	n.a.
**Hypertension**	179	29.7	n.a.
**Joint disease**	0	0.0	n.a.
**Kidney disease**	10	1.7	n.a.
**Prostate Enlargement**	6	1.0	n.a.
**Stroke**	8	1.3	n.a.
**Vision disorders**	16	2.7	n.a.
**Years of education^†^**			
0 to 6 years	92	15.3	31.2
7 to 12 years	261	43.3	27.5
≥13 years	250	41.5	41.3
**Employment status**			
Employed (full-time or part-time)	375	62.2	n.a.
Unemployed	16	2.7	n.a.
Student	33	5.5	n.a.
Full-time Homemaker	128	21.2	n.a.
Retired	44	7.3	n.a.
**Marital status^†^**			
Single	164	27.2	32.1
Married (or living as married)	411	68.2	59.6
Separated or Divorced	10	1.7	3.3
Widowed	17	2.8	4.9

*Quotas were specified for these demographic characteristics

^†^ Percentages were based on 2013 population data

n.a. not applicable

Chronic illness: medical conditions or disabilities diagnosed by a physician, that lasts a year or more, which requires ongoing medical attention or treatment and/or limits activities of daily living.

### Best-worst scaling survey result

Results (Table 4) revealed that self-care or “*being able to take care of self-care needs without the help of others*” was consistently considered the most important domain to one’s health-related quality of life (Domain #14, Total B-W score = 636, Index = 100, CV = 1.0). The following domains were likewise consistently considered important based on their relatively high index values and low CVs: healing and resistance to illness or “*not falling sick easily and getting well quickly when you are sick” (*Domain #4,Total B-W score = 461, Index = 71.4, CV = 1.6), social relationships or “*having good relationships with family*, *friends and others*” *(*Domain #26, Total B-W score = 373, Index = 64.9, CV = 2.0), physical fitness and mobility or “*being able to carry out physical activities and move around without difficulty*” *(*Domain #3,Total B-W score = 350, Index = 63.2, CV = 2.0), and personal freedom or *“being independent and in control of your life” (*Domain #18,Total B-W score = 194, Index = 50.3, CV = 4.0). The domain on sex or “*having a satisfying sex life*” was consistently considered least important (Domain #9, Total B-W score = -803, Index = 9.3, CV = 0.7). The following domains were also perceived as least important as evidenced by negative B-W scores, relatively low index scores and relatively low CVs, suggesting that these domains had a small chance of being chosen as most important: “*having normal physical appearance (acceptable weight*, *looking healthy)*” (Domain #1, Total B-W score = -461, Index = 23.1, CV = 1.7), social contact or “*interacting with others (e*.*g*. *talking*, *shared activities*, *etc*.)” (Domain #25, Total B-W score = -444, Index = 22.9, CV = 1.7), speech or “*being able to speak clearly so that others will understand (e*.*g*. *no slurring of speech)”*(Domain #13, Total B-W score = -269, Index = 26.9, CV = 2.7), and sense of growth or “*being able to improve in knowledge*, *skills*, *and emotional maturity”* (Domain #19, Total B-W score = -232, Index = 29.6, CV = 3.3).

**Table 4:** Summary of best-worst scaling survey results.

Domain #	Rank	Label	Total Count				Scaled B-W scores		Heteroge-neity		Conditional Logistic Regression		
			Best (B)	Worst (W)	B-W score	Average B-W score	Square root (B/W)	Index	SD	CV	Est	SE	Odds Ratio
**Physical Health**													
**14**	**1**	**Being able to take care of self-care needs without help from others (i.e. eating, bathing, getting dressed)**	754	118	636	1.05	2.53	100.0	1.04	1.0	0.00	—	1.00
**4**	**2**	**Not falling sick easily and getting well quickly when you are sick**	665	204	461	0.76	1.81	71.4	1.21	1.6	0.11	0.08	1.12
**3**	**3**	**Being able to carry out physical activities and move around without difficulty**	575	225	350	0.58	1.60	63.2	1.17	2.0	0.43	0.08	1.53
**11**	**4**	**Having good eyesight**	458	268	190	0.32	1.31	51.7	1.31	4.1	0.41	0.08	1.51
**2**	**5**	**Having energy to do things**	442	274	168	0.28	1.27	50.2	1.22	4.4	0.57	0.08	1.77
**15**	**6**	**No aches or pains in the body**	466	345	121	0.20	1.16	46.0	1.37	6.8	0.38	0.09	1.47
5	7	Being able to breathe well (e.g. no nose blockage, no asthma attacks)	406	298	108	0.18	1.17	46.2	1.26	7.0	0.58	0.08	1.78
10	8	Being able to sleep well	373	378	-5	-0.01	0.99	39.3	1.32	159.3	0.59	0.09	1.81
6	9	Being able to eat and digest food well	319	360	-41	-0.07	0.94	37.2	1.19	17.5	0.84	0.08	2.32
8	10	Being able to control your urine	270	364	-94	-0.16	0.86	34.1	1.23	7.9	0.84	0.08	2.32
7	11	Being able to pass motion regularly (not having constipation)	284	397	-113	-0.19	0.85	33.5	1.23	6.6	0.91	0.08	2.49
12	12	Being able to hear well	219	367	-148	-0.25	0.77	30.6	1.12	4.6	1.13	0.08	3.09
13	13	Being able to speak clearly so that others will understand (e.g. no slurring of speech)	231	500	-269	-0.45	0.68	26.9	1.18	2.7	1.05	0.09	2.87
1	14	Having normal physical appearance (acceptable weight, looking healthy)	238	699	-461	-0.76	0.58	23.1	1.33	1.7	0.75	0.10	2.11
9	15	Having a satisfying sex life	47	850	-803	-1.33	0.24	9.3	.95	0.7	1.53	0.12	4.63
**Mental Health**													
**18**	**1**	**Being independent and in control of your life**	508	314	194	0.32	1.27	50.3	1.29	4.0	0.40	0.08	1.50
**16**	**2**	**Being happy. Not sad, angry, or worried**	501	324	177	0.29	1.24	49.2	1.34	4.6	0.36	0.08	1.44
**23**	**3**	**Being mentally active (e.g. reading, learning new things, taking up a new hobby)**	450	324	126	0.21	1.18	46.6	1.18	5.7	0.67	0.08	1.96
21	4	Being able to overcome difficulties in life	454	370	84	0.14	1.11	43.8	1.28	9.2	0.55	0.08	1.74
22	5	Thinking positively in life	457	386	71	0.12	1.09	43.0	1.37	11.6	0.48	0.08	1.61
24	6	Having a good memory (able to remember objects, thoughts, and events)	433	388	45	0.07	1.06	41.8	1.29	17.3	0.66	0.08	1.93
17	7	Having confidence in yourself	345	412	-67	-0.11	0.92	36.2	1.24	11.2	0.80	0.08	2.22
20	8	Being able to find satisfaction and meaning in the things that you do	338	503	-165	-0.27	0.82	32.4	1.28	4.7	0.78	0.09	2.18
19	9	Being able to improve in knowledge, skills, and emotional maturity	295	527	-232	-0.38	0.75	29.6	1.26	3.3	0.83	0.09	2.30
**Social Health**													
**26**	**1**	**Having good relationships with family, friends and others**	594	221	373	0.62	1.64	64.9	1.25	2.0	0.26	0.08	1.30
27	2	Having a role in the lives of others and being able to fulfil that role (e.g. being a good employee, a good wife, a good son)	395	354	41	0.07	1.06	41.8	1.30	19.1	0.65	0.08	1.92
25	3	Interacting with others (e.g. talking, shared activities, etc.)	225	669	-444	-0.74	0.58	22.9	1.23	1.7	0.91	0.10	2.49

SD, standard deviation of total B-W score; CV, coefficient of variation; Est, estimate; SE, standard error. **Items in bold font among the 10 highest-ranked domains.**

In general, the health domains found at the extremes of the importance continuum at the population level (i.e., the most and least important) were reproduced at the subgroup levels (see Tables 5 and 6). This was most evident in the domains of “*self-care*” and “*having a satisfying sex life*” which were ranked respectively as most important (i.e., highest index and very low CV values) and least important (i.e., lowest index and very low CV values) across all subgroups of gender, ethnicity, age groups, presence of chronic illness, and language of interview. The following domains were consistently retained among the most important across the subgroups: Domain #4 “*not falling sick easily and getting well quickly when you are sick*”, Domain #26 “*having good relationships with family*, *friends and others*”, and Domain #3 “*being able to carry out physical activities and move around without any difficulty*”. The following domains were consistently retained among the least important across subgroups: Domain #1 “*having a normal physical appearance*”, Domain #25 “*interacting with others (e*.*g*. *talking*, *shared activities*, *etc*.)”, and Domain #13 “*being able to speak clearly so that others will understand (e*.*g*. *no slurring of speech)*”.

**Table 5:** Importance ranks (index values, IV) and consistency of ranks (coefficient of variation, CV) by gender and age group.

Health Domain	Label	Gender IV (CV)		Age IV (CV)		
		Male	Female	21–34 Years	35–49 Years	50 & up Years
**Physical Health**						
1	Having normal physical appearance	25.1 (1.77)	21.1 (1.71)	27.9 (1.85)	23.5 (1.82)	19.0 (1.61)
2	Having energy to do things	53.5 (4.32)	47.1 (4.42)	56.4 (5.32)	47.8 (5.66)	47.1 (3.17)
3	Being able to carry out physical activities and move around without difficulty	65.6 (2.08)	61.0 (1.95)	67.1 (2.58)	61.8 (2.14)	60.9 (1.59)
4	Not falling sick easily and getting well quickly when you are sick	77.1 (1.60)	66.1 (1.56)	75.1 (1.90)	77.8 (1.40)	64.3 (1.47)
5	Being able to breathe well	47.5 (9.29)	44.7 (5.70)	59.6 (4.19)	43.1 (13.63)	38.5 (8.71)
6	Being able to eat and digest food well	38.5 (11.52)	35.9 (35.87)	41.1 (9.15)	36.8 (14.07)	33.9 ()
7	Being able to pass motion regularly	35.0 (5.93)	31.9 (7.29)	33.0 (3.13)	33.6 (6.63)	33.6 (136.07)
8	Being able to control your urine	32.6 (4.74)	34.9 (19.36)	33.6 (3.80)	33.9 (7.49)	33.5 (94.71)
9	Having a satisfying sex life	12.9 (0.90)	5.9 (0.55)	10.3 (0.75)	12.5 (0.89)	5.8 (0.56)
10	Being able to sleep well	43.0 (55.75)	35.9 (33.91)	38.5 (5.74)	39.0 (59.30)	40.3 (6.14)
11	Having good eyesight	59.5 (3.23)	45.4 (5.43)	46.1 (262.02)	47.4 (6.41)	63.8 (1.77)
12	Being able to hear well	29.9 (3.60)	30.7 (6.06)	32.3 (3.44)	28.2 (3.23)	30.4 (10.79)
13	Being able to speak clearly so that others will understand	29.1 (2.70)	24.8 (2.61)	28.9 (2.34)	23.6 (1.95)	27.0 (4.23)
14	Being able to take care of self-care needs without help from others (e.g., eating, bathing, getting dressed)	100.0 (1.07)	100.0 (0.91)	100.0 (1.27)	100.0 (0.99)	100.0 (0.77)
15	No aches or pains in the body	48.1 (7.70)	43.9 (6.13)	42.2 (11.34)	47.1 (6.08)	48.3 (2.76)
**Mental Health**						
16	Being happy. Not sad, angry or worried	48.0 (7.37)	50.3 (3.33)	56.5 (5.14)	55.6 (2.96)	39.3 (6.52)
17	Having confidence in yourself	36.3 (6.48)	36.0 (35.12)	43.2 (14.29)	38.5 (31.50)	29.1 (6.48)
18	Being independent and in control of your life	55.7 (3.39)	45.4 (4.81)	64.3 (2.90)	47.3 (5.71)	41.9 (4.45)
19	Being able to improve in knowledge, skills and emotional maturity	32.0 (3.41)	27.3 (3.16)	48.3 (22.15)	31.1 (3.98)	17.5 (1.38)
20	Being able to find satisfaction and meaning in the things that you do	34.0 (4.30)	30.8 (5.13)	47.2 (51.48)	33.7 (5.60)	21.7 (2.10)
21	Being able to overcome difficulties in life	46.7 (9.30)	41.0 (9.06)	58.4 (4.01)	50.1 (3.99)	30.2 (8.08)
22	Thinking positively in life	46.0 (10.81)	40.1 (12.50)	56.8 (4.76)	49.7 (4.24)	29.2 (6.70)
23	Being mentally active	51.1 (4.85)	42.5 (6.73)	59.9 (3.45)	47.1 (5.69)	36.5 (12.82)
24	Having good memory	45.3 (13.02)	38.5 (24.99)	44.8 (28.76)	35.2 (8.03)	44.3 (3.44)
**Social Health**						
25	Interacting with others (talking, shared activities, etc)	24.4 (1.69)	21.5 (1.66)	29.0 (1.94)	20.2 (1.35)	20.1 (1.76)
26	Having good relationships with family, friends and others	70.7 (1.89)	59.4 (2.16)	77.6 (1.94)	80.3 (1.46)	48.1 (2.80)
27	Having a role in the lives of others and being able to fulfil that role	46.6 (10.50)	37.4 (98.88)	54.5 (6.48)	42.0 (18.08)	32.7 (30.16)

**Table 6:** Importance ranks (index values, IV) and consistency of ranks (coefficient of variation, CV) by ethnicity and presence of chronic conditions.

Health Domain	Label	Ethnicity Index Values (CV)			Chronic condition Index Values (CV)	
		Chinese	Malay	Indian	Present	Absent
**Physical Health**						
1	Having normal physical appearance (acceptable weight, looking healthy)	18.4 (1.32)	26.6 (2.40)	29.9 (2.43)	20.7 (1.70)	25.6 (1.78)
2	Having energy to do things	46.3 (4.82)	49.1 (4.77)	59.8 (3.45)	47.3 (3.75)	53.3 (5.17)
3	Being able to carry out physical activities and move around without difficulty	65.0 (1.73)	58.8 (2.25)	64.7 (2.50)	60.6 (1.78)	66.1 (2.28)
4	Not falling sick easily and getting well quickly when you are sick	80.7 (1.18)	58.7 (2.19)	71.1 (2.17)	71.6 (1.35)	72.0 (1.86)
5	Being able to breathe well (e.g. no nose blockage, no asthma attacks)	45.8 (5.33)	39.8 (60.33)	53.5 (5.80)	37.1 (30.02)	57.7 (3.83)
6	Being able to eat and digest food well	35.0 (15.77)	33.9 (7.56)	45.6 (37.42)	35.6 (173.78)	39.0 (9.43)
7	Being able to pass motion regularly(not having constipation)	33.9 (11.40)	25.4 (2.42)	41.9 (19.77)	34.1 (23.58)	33.0 (3.90)
8	Being able to control your urine	38.0 (74.13)	25.7 (2.97)	34.8 (4.66)	34.1 (23.22)	33.7 (4.69)
9	Having a satisfying sex life	8.7 (0.68)	8.3 (0.67)	11.6 (0.86)	8.1 (0.65)	10.7 (0.78)
10	Being able to sleep well	41.8 (9.70)	29.5 (3.57)	45.5 (41.75)	40.6 (8.44)	38.1 (7.90)
11	Having good eyesight	50.9 (3.59)	44.7 (8.64)	61.0 (3.39)	58.2 (2.33)	46.9 (14.68)
12	Being able to hear well	29.5 (5.15)	28.9 (3.97)	34.1 (4.19)	29.5 (6.10)	31.3 (3.56)
13	Being able to speak clearly so that others will understand (e.g. no slurring of speech)	24.6 (2.52)	28.8 (3.20)	29.5 (2.51)	25.9 (3.04)	27.5 (2.35)
14	Being able to take care of self-care needs without help from others (i.e. eating, bathing, getting dressed)	100.0 (0.94)	100.0 (0.92)	100.0 (1.14)	100.0 (0.83)	100.0 (1.16)
15	No aches or pains in the body	53.5 (2.71)	35.0 (10.01)	42.8 (29.71)	48.3 (3.36)	43.8 (209.61)
**Mental Health**						
16	Being happy. Not sad, angry, or worried	50.4 (3.50)	43.7 (8.50)	53.5 (5.06)	43.8 (4.72)	55.0 (4.41)
17	Having confidence in yourself	30.3 (4.58)	40.3 (32.11)	46.3 (22.70)	31.7 (8.14)	41.1 (17.45)
18	Being independent and in control of your life	45.3 (5.16)	56.1 (2.57)	55.3 (4.29)	43.7 (4.74)	57.6 (3.47)
19	Being able to improve in knowledge, skills, and emotional maturity	25.5 (2.50)	33.0 (5.50)	34.6 (3.87)	21.5 (1.83)	39.7 (10.23)
20	Being able to find satisfaction and meaning in the things that you do	31.5 (5.52)	31.4 (4.10)	35.0 (3.97)	24.5 (2.52)	41.9 (22.90)
21	Being able to overcome difficulties in life	41.6 (9.06)	44.9 (6.64)	47.1 (15.04)	35.1 (49.14)	54.8 (4.03)
22	Thinking positively in life	37.7 (134.73)	51.0 (3.62)	47.1 (16.70)	34.8 (34.01)	52.9 (4.95)
23	Being mentally active (e.g. reading, learning new things, taking up a new hobby)	46.1 (4.35)	42.1 (12.52)	52.7 (5.64)	40.7 (7.02)	53.1 (4.80)
24	Having a good memory (able to remember objects, thoughts, and events)	34.7 (12.51)	56.9 (2.45)	44.6 (192.02)	42.3 (5.62)	41.3 (17.98)
**Social Health**						
25	Interacting with others (e.g. talking, shared activities, etc.)	19.9 (1.37)	27.9 (2.86)	24.5 (1.58)	20.3 (1.62)	25.8 (1.73)
26	Having good relationships with family, friends and others	55.9 (2.51)	74.6 (1.44)	74.4 (2.00)	53.8 (2.48)	78.2 (1.68)
27	Having a role in the lives of others and being able to fulfil that role (e.g. being a good employee, a good wife, a good son)	35.1 (17.25)	44.9 (6.87)	53.9 (5.32)	35.7 (394.37)	48.7 (9.27)

The sensitivity analysis showed an agreement between the ranking approaches based on simple summary statistics and the exploded logit model in the domains that were perceived as most and least important (Table 3, “Est” and “Odds ratio” columns). The most important domains, those with the highest total B-W scores (or highest index values) were associated with lowest beta estimates or odds ratios. We found a strong negative correlation between the total B-W scores and the beta estimates (r = -0.91).

The proportion of participants who were unable to rank more than three choice tasks (out of 18) was 6.1%. The most prominent reasons were that the domains in the choice task were either all equally important (4.3%) or not comparable (4.1%). Given that the five top-ranked domains were consistent across subgroups and the clear (dominant) preferences at the aggregate level (as seen by the separation of the BW scores between domains in the top tertile compared with the middle or lowest tertile), we believe that the small proportion of participants who were unable to rank more than three choice tasks did not affect the overall rankings.

## Discussion

We sought to determine which of the different aspects of health Singaporeans perceived to be the most important to their quality of life using domains from an HRQOL framework developed from the ground-up. We found that the participants consistently ranked the following domains as the most important aspects of health: self-care, healing and resistance to illness, social relationships, physical fitness and mobility, and emotions, and that the highest and lowest ranked domains were consistent for age, gender, ethnicity, presence of chronic illness, and language of interview. To the best of our knowledge, this is the first such study in Asia ranking the importance of the different aspects of health in the general population using best-worst scaling, which minimizes variations in the way participants interpret the meaning of scales and response options, thereby ensuring accurate measurements of item ranks.

Globally, studies that rank different aspects of health are few and far between, and our data add to the literature on this topic. Our results align with other importance-hierarchy studies done worldwide by the WHO, and in Japan and Great Britain; this suggests that the highest ranked domains of health may be similar in various socio-cultural contexts. An analysis of the importance-hierarchy of life aspects was carried out using the data collected during the field trial of the WHOQOL group in the 1990s. In the field trial, 4804 respondents across 15 centers worldwide (seven developed countries, eight developing countries) were asked to indicate how important each of a list of life aspects were and how much it affected their QOL using a 5-point Likert scale with descriptors that ranged from “not important” to “extremely important”. The most important aspects of life in the overall analysis, and for the centers in developed countries (United Kingdom, Spain, Australia, France, United States, Netherlands, and Japan), were daily living activities, having energy, overall health, happiness and enjoyment of life, and to move around [33]. The WHOQOL-BREF importance items on daily living activities, happiness and enjoyment of life, and to move around corresponded with our own items of self-care, emotions, and physical fitness and mobility respectively.

Using methods similar to the previous WHOQOL study, the WHOQOL measures of quality of life for use with older adults (WHOQOL-OLD) pilot study, which recruited 7401 adults, 60 years and older across 22 countries, provided further information on the importance-hierarchy of the WHOQOL facets. Among the countries studied were three urban populations in Asia: Tokyo, Japan; Guangzhou, China; and Hong Kong [34]. The most important aspects of life in these urban populations were: overall health, freedom from pain, energy, restful sleep, activities of daily living, availability of medicines and treatment, sensory abilities, and autonomy [35]. A survey conducted in Japan in 1997 (n = 1096) asked respondents to 1) rank (from 1 to 10) each of the ten pre-selected quality of life domains according to the order of importance in their lives and then 2) provide importance scores to each of the items on a scale of a hundred. This study showed that respondents valued personal health and relationship with family as the most important [36]. A survey that recruited 2033 individuals from representative households in Great Britain in 1992 asked participants to spontaneously mention five “most important things in their current life (both good and bad)”; participants were then asked to rank order the items according to importance. This study showed that respondents were most likely to freely mention relationships with family or relatives, their own health, the health of a close person, and finances/standard of living/housing (in that order) [37].

Although previous surveys have shown that personal health is among the most important aspects perceived to contribute to quality of life across many countries, including Singapore [38, 39], they do not provide insight on which specific aspects of perceived health were thought to be most important. In surveys conducted across almost two decades, Singaporeans have consistently chosen health as the one thing they wanted most [39, 40]. Identifying the specific aspects of health that Singaporeans value the most, and targeting improvement in those aspects of health, could potentially lead to greater improvements in HRQOL. In addition, surveys have shown that personal health and family, along with having a comfortable home and a job, were the aspects of life most valued by Singaporeans [39]. Three of the five highest ranked domains in our study were items relating to physical health; our item for social relationships, defined as “having good relationships with family, friends and others”, includes family relationships.

Sex, which was defined as “having a satisfying sex life” in our study, was consistently selected as the least important of the 27 domains ranked in this study. This is consistent with the results of the WHOQOL-BREF study which showed that “sexual life” was consistently ranked the lowest, be it in the overall analysis, the analysis using data from developed countries, or the analysis using data from developing countries (Zimbabwe, Thailand, India (two centers), Panama, Israel, Croatia, Russia) [33]. Interestingly, respondents from six of the seven developed countries in the WHOQOL-BREF ranked “sexual life” as the least important of 25 items of health [33]. Given that our ranking survey was interviewer administered, it is possible that respondents, members of a conservative, Asian society, may have been wary of selecting “having a satisfying sex life” as the most important domain. However, the majority of respondents in the WHOQOL-BREF completed the questionnaire unassisted, making socially-desirable responses less likely. Despite this, participants in the WHOQOL-BREF still ranked “sexual life” as the item with the lowest importance to their life. This would seem to support that sexual life may truly be of low priority for the surveyed respondents.

The majority of studies determined the importance-hierarchy of quality of life domains by using simple rank ordering (i.e., of X items, rank each one in order of importance from 1 to X) or by assigning scores to each of the proffered domains, either through a categorical (e.g. Likert scale) or continuous scale. There is considerable cognitive burden with the former which thus limits the number of items that a given participant can consider at any one time. The latter, on the other hand, is subject to the influence of culturally-dependent differences in the interpretation of rating scales and/or personal response styles [18–20] or the risk of acquiescent response bias. By inquiring on the extremes of a latent construct, BWS overcomes these stated limitations and minimizes the chance that a number of objects are perceived as all “extremely important” [21,23].

We recognize several limitations of this study. First, we used object-case (Case 1) best-worst scaling to establish which aspects of health are most important to Singaporeans. The robust method allowed us to present a large number of items, 27 domains, without overwhelming respondents with a high cognitive burden. By presenting groups of only three domains at any one time, we increased the likelihood that respondents were able to provide us meaningful, thought out responses. However, the method also meant that respondents were not able to consider all 27 domains together and make a conscious decision on how each item is ranked relative to all the other items. Second, the BIBD design we implemented allowed us to establish item ranks across the entire sampled population but not on a “per individual” level.

Flynn has spoken of numerous potential applications of object-case BWS to establish relative importance in healthcare [23]. However, to our knowledge, only one other study used an object-case BWS exercise to establish both the relative importance and the magnitude of the importance across different aspects of health. This study used object-case BWS to establish the importance of different aspects of activities of daily living in the Disability Assessment for Dementia (DAD) scale as reported by the caregivers of patients with Alzheimer’s Disease (AD) (n = 403 (USA), n = 400 (Germany). The study found that the caregivers of patients with AD valued preserved basic activities of daily living (e.g., using the toilet without accidents) over instrumental activities of daily living (e.g., handling money) [24].

## Conclusion

We found that respondents in our survey consistently ranked self-care, healing and resistance to illness, social relationships, physical fitness and mobility, and emotions as the most important aspects of health. Our results are similar to those from the few previous studies in this area, suggesting that the highest ranked domains of health may be similar in various socio-cultural contexts.

## Supporting information

S1 Table Design of best-worst scaling choice sets (13 versions). (DOCX)

S1 Fig BWS sample show card. (DOCX)
