# Supplementary material for: Using best-worst scaling choice experiments to elicit the most important domains of health for health-related quality of life in Singapore
Source: PLoS One. 2018 Feb 8;13(2):e0189687. doi: 10.1371/journal.pone.0189687 (PMC5805165; doi:10.1371/journal.pone.0189687)
Supplement: S1 Table — (DOCX) [file pone.0189687.s001.docx]

**S1 Table. Design of best-worst scaling choice sets (13 versions).**

| **Version** | **Choice_set** | **Domain_1** | **Domain_2** | **Domain_3** |
| --- | --- | --- | --- | --- |
| **1** | **1** | **1** | **2** | **3** |
| **1** | **2** | **4** | **5** | **6** |
| **1** | **3** | **7** | **8** | **9** |
| **1** | **4** | **10** | **11** | **12** |
| **1** | **5** | **13** | **14** | **15** |
| **1** | **6** | **16** | **17** | **18** |
| **1** | **7** | **19** | **20** | **21** |
| **1** | **8** | **22** | **23** | **24** |
| **1** | **9** | **25** | **26** | **27** |
| **2** | **1** | **7** | **1** | **4** |
| **2** | **2** | **8** | **2** | **5** |
| **2** | **3** | **9** | **3** | **6** |
| **2** | **4** | **16** | **10** | **13** |
| **2** | **5** | **17** | **11** | **14** |
| **2** | **6** | **18** | **12** | **15** |
| **2** | **7** | **25** | **19** | **22** |
| **2** | **8** | **26** | **20** | **23** |
| **2** | **9** | **27** | **21** | **24** |
| **3** | **1** | **5** | **9** | **1** |
| **3** | **2** | **6** | **7** | **2** |
| **3** | **3** | **4** | **8** | **3** |
| **3** | **4** | **14** | **18** | **10** |
| **3** | **5** | **15** | **16** | **11** |
| **3** | **6** | **13** | **17** | **12** |
| **3** | **7** | **23** | **27** | **19** |
| **3** | **8** | **24** | **25** | **20** |
| **3** | **9** | **22** | **26** | **21** |
| **4** | **1** | **1** | **6** | **8** |
| **4** | **2** | **2** | **4** | **9** |
| **4** | **3** | **3** | **5** | **7** |
| **4** | **4** | **10** | **15** | **17** |
| **4** | **5** | **11** | **13** | **18** |
| **4** | **6** | **12** | **14** | **16** |
| **4** | **7** | **19** | **24** | **26** |
| **4** | **8** | **20** | **22** | **27** |
| **4** | **9** | **21** | **23** | **25** |
| **5** | **1** | **19** | **1** | **10** |
| **5** | **2** | **20** | **2** | **11** |
| **5** | **3** | **21** | **3** | **12** |
| **5** | **4** | **22** | **4** | **13** |
| **5** | **5** | **23** | **5** | **14** |
| **5** | **6** | **24** | **6** | **15** |
| **5** | **7** | **25** | **7** | **16** |
| **5** | **8** | **26** | **8** | **17** |
| **5** | **9** | **27** | **9** | **18** |
| **6** | **1** | **11** | **21** | **1** |
| **6** | **2** | **12** | **19** | **2** |
| **6** | **3** | **10** | **20** | **3** |
| **6** | **4** | **14** | **24** | **4** |
| **6** | **5** | **15** | **22** | **5** |
| **6** | **6** | **13** | **23** | **6** |
| **6** | **7** | **17** | **27** | **7** |
| **6** | **8** | **18** | **25** | **8** |
| **6** | **9** | **16** | **26** | **9** |
| **7** | **1** | **1** | **12** | **20** |
| **7** | **2** | **2** | **10** | **21** |
| **7** | **3** | **3** | **11** | **19** |
| **7** | **4** | **4** | **15** | **23** |
| **7** | **5** | **5** | **13** | **24** |
| **7** | **6** | **6** | **14** | **22** |
| **7** | **7** | **7** | **18** | **26** |
| **7** | **8** | **8** | **16** | **27** |
| **7** | **9** | **9** | **17** | **25** |
| **8** | **1** | **25** | **1** | **13** |
| **8** | **2** | **26** | **2** | **14** |
| **8** | **3** | **27** | **3** | **15** |
| **8** | **4** | **19** | **4** | **16** |
| **8** | **5** | **20** | **5** | **17** |
| **8** | **6** | **21** | **6** | **18** |
| **8** | **7** | **22** | **7** | **10** |
| **8** | **8** | **23** | **8** | **11** |
| **8** | **9** | **24** | **9** | **12** |
| **9** | **1** | **14** | **27** | **1** |
| **9** | **2** | **15** | **25** | **2** |
| **9** | **3** | **13** | **26** | **3** |
| **9** | **4** | **17** | **21** | **4** |
| **9** | **5** | **18** | **19** | **5** |
| **9** | **6** | **16** | **20** | **6** |
| **9** | **7** | **11** | **24** | **7** |
| **9** | **8** | **12** | **22** | **8** |
| **9** | **9** | **10** | **23** | **9** |
| **10** | **1** | **1** | **15** | **26** |
| **10** | **2** | **2** | **13** | **27** |
| **10** | **3** | **3** | **14** | **25** |
| **10** | **4** | **4** | **18** | **20** |
| **10** | **5** | **5** | **16** | **21** |
| **10** | **6** | **6** | **17** | **19** |
| **10** | **7** | **7** | **12** | **23** |
| **10** | **8** | **8** | **10** | **24** |
| **10** | **9** | **9** | **11** | **22** |
| **11** | **1** | **22** | **1** | **16** |
| **11** | **2** | **23** | **2** | **17** |
| **11** | **3** | **24** | **3** | **18** |
| **11** | **4** | **25** | **4** | **10** |
| **11** | **5** | **26** | **5** | **11** |
| **11** | **6** | **27** | **6** | **12** |
| **11** | **7** | **19** | **7** | **13** |
| **11** | **8** | **20** | **8** | **14** |
| **11** | **9** | **21** | **9** | **15** |
| **12** | **1** | **17** | **24** | **1** |
| **12** | **2** | **18** | **22** | **2** |
| **12** | **3** | **16** | **23** | **3** |
| **12** | **4** | **11** | **27** | **4** |
| **12** | **5** | **12** | **25** | **5** |
| **12** | **6** | **10** | **26** | **6** |
| **12** | **7** | **14** | **21** | **7** |
| **12** | **8** | **15** | **19** | **8** |
| **12** | **9** | **13** | **20** | **9** |
| **13** | **1** | **1** | **18** | **23** |
| **13** | **2** | **2** | **16** | **24** |
| **13** | **3** | **3** | **17** | **22** |
| **13** | **4** | **4** | **12** | **26** |
| **13** | **5** | **5** | **10** | **27** |
| **13** | **6** | **6** | **11** | **25** |
| **13** | **7** | **7** | **15** | **20** |
| **13** | **8** | **8** | **13** | **21** |
| **13** | **9** | **9** | **14** | **19** |
