# Supplementary figures and images for: Using best-worst scaling choice experiments to elicit the most important domains of health for health-related quality of life in Singapore
Source: PLoS One. 2018 Feb 8;13(2):e0189687. doi: 10.1371/journal.pone.0189687 (PMC5805165; doi:10.1371/journal.pone.0189687)

**S1 Fig. BWS sample show card.**


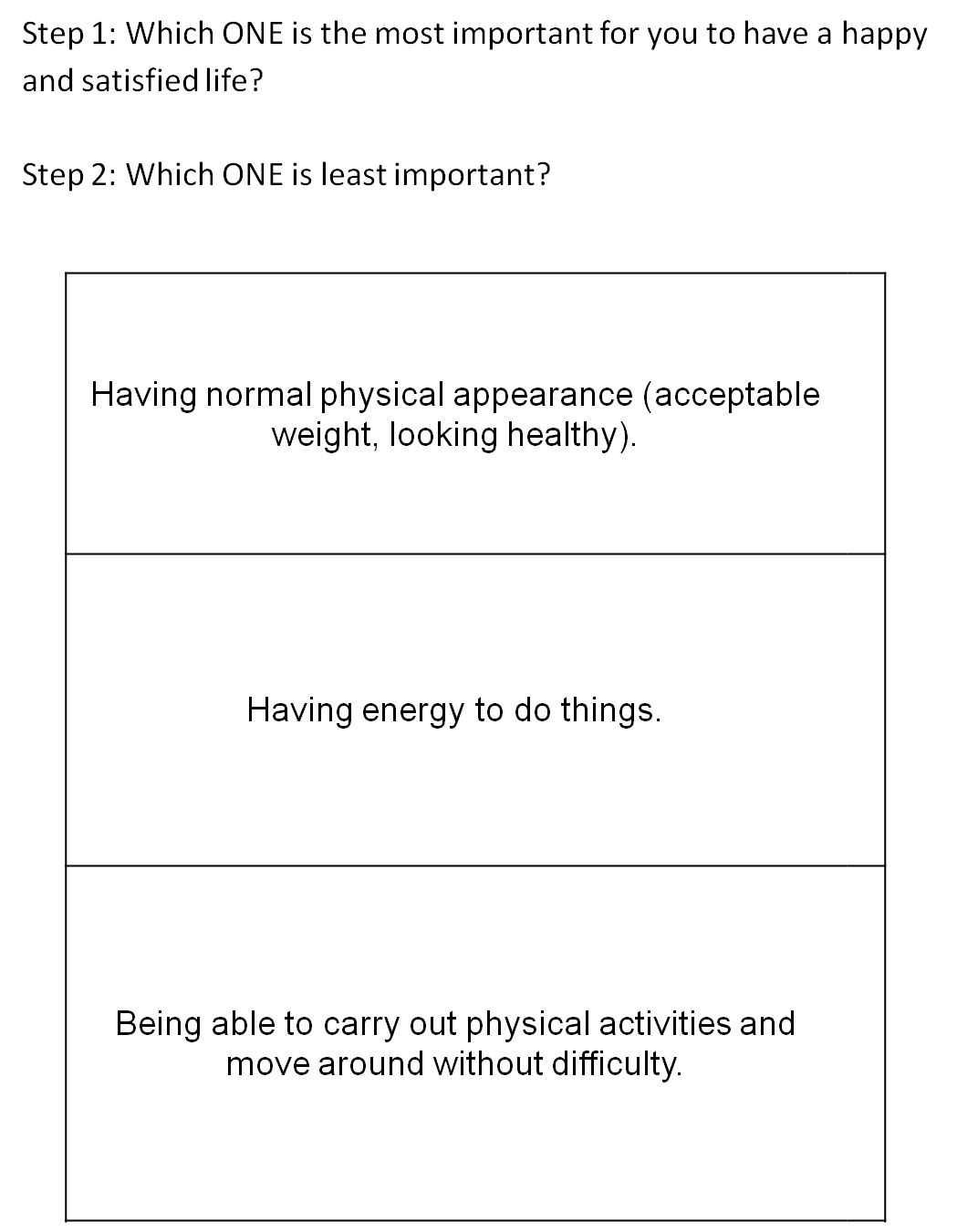

Supplement: S1 Fig — (DOCX) [file pone.0189687.s002.docx]
